# Cross-cultural validation of the profile of mood scale: evaluation of the psychometric properties of short screening versions

**DOI:** 10.3389/fpsyg.2025.1498717

**Published:** 2025-03-17

**Authors:** Ileana Schmalbach, Bjarne Schmalbach, Alireza Aghababa, Ralf Brand, Yu-Kai Chang, Muhammet Cihat Çiftçi, Hassan Elsangedy, Jesús Fernández Gavira, Zhijian Huang, Hafrún Kristjánsdóttir, Luca Mallia, Sanaz Nosrat, Caterina Pesce, Daði Rafnsson, Daniel Medina Rebollo, Sinika Timme, Elmar Brähler, Katja Petrowski

**Affiliations:** ^1^Department of Medical Psychology and Medical Sociology, Johannes-Gutenberg University Mainz, Mainz, Germany; ^2^Department of Sport Psychology, Sport Sciences Research Institute of Iran, Tehran, Iran; ^3^Sport and Exercise Psychology, University of Potsdam, Potsdam, Germany; ^4^Department of Physical Education and Sport Sciences, National Taiwan Normal University, Taipei, Taiwan; ^5^Department of Sport Sciences, Ankara Yıldırım Beyazıt University, Ankara, Türkiye; ^6^Department of Physical Education, Federal University of Rio Grande do Norte, Natal, Brazil; ^7^Department of Physical Education and Sports, University of Seville, Seville, Spain; ^8^Department of Physical Education, Hubei University, Wuhan, China; ^9^Department of Sport Science, Reykjavik University, Reykjavik, Iceland; ^10^Department of Movement, Human and Health Sciences, University of Rome Foro Italico, Rome, Italy; ^11^Department of Health Sciences, Lehman College, City University of New York, New York, NY, United States; ^12^Department of Medical Psychology and Medical Sociology, University Medical Center of Leipzig, Leipzig, Germany

**Keywords:** mood, affect, survey research, psychometric evaluation, confirmatory factor analysis

## Abstract

The Profile of Mood States (POMS) is one of the most widely applied scales for measuring mood. Considering the advantages of short scales and increased international research, the aim of the present study was to evaluate cross-culturally the psychometric properties of a short 16-item version of the POMS. Data were collected from 15,693 participants across 10 different countries worldwide. Initially, we identified the original versions of the POMS in various languages. Subsequently, we selected 16 items based on the previously validated short form (POMS-16) for analysis. Psychometric properties of the POMS were then evaluated in samples from each studied population for each language version. Confirmatory factor analysis was conducted to assess its invariance across age groups and gender, alongside reliability estimation. Most language versions of the POMS-16 showed a good fit with the four-factor model, except for the Chinese (traditional) and Turkish versions. Reliability was generally high, except for the *Vigor* subscale in a small subset of languages. Regarding measurement invariance, the majority of language versions were invariant across gender and age groups, except for the Farsi language version across gender, and the Chinese, Farsi, Finnish, and Turkish versions across age. These findings enhance the cross-cultural applicability of the POMS-16, contributing to its utility in diverse populations and thus enhancing the comparability of the results. In addition, we introduced the first versions of the POMS in Farsi, Finnish, and Icelandic.

## Background

“Mood matters”, posited Lane and Terry (2000) – both, for basic and applied psychological research. Moods are mild and pervasive affective states (McNair et al., 1971) influenced by psychophysiological responses (Soylu, 2021) that significantly impact wellbeing, influencing behavioral patterns and perception (e.g., perceived health outcomes; Berger et al., 1998). Lane and Terry (2000) proposed a general definition of mood being “a set of feelings, ephemeral in nature, varying in intensity and duration, and usually involving more than one emotion” (Lane and Terry, 2000, p. 7). A pivotal factor in this description is that mood and emotion are understood as part of the same theoretical background, as making a definitive distinction between them remains a subject of debate (DeLancey, 2006, pp. 527–538; Lane et al., 2005). At a specific level, it is posited that mood encompasses an evaluative facet, namely the extent to which mood is perceived as pleasant, coupled with an arousal component, characterized by varying degrees of activity (Terry and Lane, 2000).

Mood impacts cognitive performance at a basic level (Schwarz and Clore, 2003) but is also relevant in collaborative settings, e.g., via mood contagion (Jordan et al., 2006; Neumann and Strack, 2000). Through these pathways, mood plays an important role in various contexts, such as the workplace (Morfeld et al., 2007; Selmi et al., 2023) and athletic performance (Aydi et al., 2022). In the clinical and psychotherapeutic context, the assessment of mood states is crucial for understanding and addressing mental health concerns (e.g., monitoring mood fluctuations diagnostics, treatment evaluation; Classen et al., 2001; Grulke et al., 2004; Hosaka et al., 2001).

A quick history of the Profile of Mood States (POMS) reflects the amount of effort put into measuring mood state (POMS; McNair et al., 1971). In its original form (McNair et al., 1971, 1992), it includes 65 items that are loaded on 7 different scales: depression, anxiety, fatigue, vigor, irritability, tension, and confusion. Initially, seven items constituted a *Friendliness* factor, which was excluded due to poor discriminant validity with the *Vigor-Activity* factor. However, few adaptations of the POMS retained this component (Andrade et al., 2010). The intensity of the mood is rated on a 5-point Likert scale ranging from “0 = not at all” to “4 = very strong.” Commonly used time frames reflecting mood over a specific period of time include: Today, Right Now, and This Week (e.g., “How did you feel today?” vs. “the last week including today; Gibson, 1997). The total score (total mood disturbance) is calculated by subtracting the (positive) value of the *Vigor* subscale from the sum of the remaining scales. However, different scoring procedures are described with regard to the calculation of scale values (Kieviet-Stijnen et al., 2008). It consistently achieved high internal consistency (*α* of 0.84–0.95; McNair et al., 1971, 1992) and its construct validity is supported by past research (e.g., Morris and Salmon, 1994; Watson and Clark, 1992).

Past studies examining its factor structure provided substantial evidence for most of the seven factors (Norcross et al., 1984), with the exception of the *Confusion* subscale (Bourgeois et al., 2012; Morfeld et al., 2007; Netz et al., 2005), which was instead regarded as a cognitive state (Lane et al., 2007). POMS is one of the most widely used questionnaires providing several advantages. First, it is a multidimensional self-report instrument that captures the transient and oscillating nature of mood states (McNair et al., 1971, 1992). Furthermore, it is a versatile tool that can be applied in a variety of settings extending from the psychotherapeutic and medical field (e.g., Baker et al., 2002; Braslis et al., 1995; Gross, 1991; López-Jiménez et al., 2021; Szaflarski et al., 2003; von Steinbüchel et al., 1994) to sport psychology (Leunes and Burger, 2000; Lochbaum et al., 2021). Moreover, it has been widely applied and validated in several languages (Chinese; Cheung, 1999; Cheung and Lam, 2005; German; Grulke et al., 2006; Italian; Mannarini et al., 2012; Peri et al., 2000; Portuguese, Spanish; Andrade et al., 2013; Fernández et al., 2000; Perczek et al., 2000, Turkish; Selvi et al., 2011). Due to increased international research and the need for outcome comparability, cross-cultural validation of a scale is paramount. In addition, considering that affective states may be influenced by sociological and cultural aspects, measuring mood states across cultures may provide important insights into universal aspects of mood in a variety of settings.

The German version of the POMS (Biehl and Landauer, 1975; Biehl et al., 1986) presented the first psychometrical analysis, with satisfactory psychometric results (*a* = 0.88–0.94). A short version with 35 items (Bullinger et al., 1990) indicated satisfying factorial validity and internal consistency (*α* = 0.90); however, the data were based on a student sample (Bullinger et al., 1990; Gross, 1991). A replication in a larger, population-representative sample demonstrated similarly satisfactory internal consistency (*a* = 0.89–0.95), although it still revealed a limited factorial structure (Albani et al., 2005). Due to the limitations in the factorial structure, Petrowski et al. (2021) aimed to empirically identify a shorter version of the POMS with improved factorial validity. A psychometrically optimal 16-item solution among all valid combinations of the full POMS was found with a four-factorial structure and very good psychometric properties (*a* = 0.86–0.91). This version is strictly invariant across age groups and shows strong and partial strict invariance across genders. It represents the shortest version of the POMS available, aside from the English POMS (Cella et al., 1987; 11-items). However, the latter only provides a total mood disturbance score without subscales or norms, emphasizing the uniqueness of the German POMS-16 and the developmental lack in other languages.

Ease of administration is an advantage when collecting data, especially when considering target populations in clinical contexts (e.g., patients with cancer and chronic pain) and in epidemiological research. The importance of using brief instruments with robust psychometric properties cannot be overstated. Therefore, preventing exhaustion, resistance, and boredom during the completion of the questionnaire is key. The purpose of the present study was thus to provide a brief measure of mood. To this end, we evaluated the psychometric properties of a short version of the POMS-16 in 11 languages. In this study, we examined whether the described four-factorial structure of the instrument could be replicated and explored additional characteristics of the scales, such as measurement invariances across age and gender.

## Method

This study used a cross-sectional design to investigate mood, and its data were collected during the initial COVID lockdowns, between 29 March 2020 and 7 May 2020. The various language versions of the POMS were used as part of a separate project (Brand et al., 2020) by the International Research Group on COVID and Exercise (IRG). Existing versions of the POMS in various languages were used where available. In cases where no translation was available, the items were translated by bilingual experts in the field of study and subsequently independently checked for the quality of the translation. The translations were based on the English version of the POMS-16. Participants were informed about study procedures, data collection, and anonymization of personal data, and provided informed consent as required by German law, documented prior to commencement of the survey. Participants were recruited either through the research team’s personal networks or by responding to invitations shared via mailing lists and social media platforms, which provided a link to the online questionnaire.

### Instruments

*Profile of Mood Scales* (POMS-16; Petrowski et al., 2021). In the study at hand, we implemented a recently validated short version (POMS-16) based on the original long-form POMS-65 (McNair et al., 1971, 1992). The items are grouped into four factors: dejection, vigor, fatigue, and anger. The intensity of the mood is rated on a 5-point Likert scale ranging from “0 = not at all” to “4 = very strong,” reflecting mood over a specific period of time (“How have you felt during the past week including today?”). The scores of each individual subscale range from 0 to 16.

### Statistical analysis

All analyses were conducted in *R*, using the packages *lavaan* and *semTools* (Rosseel, 2012; Jorgensen et al., 2019). Specifically, we conducted confirmatory factor analysis (CFA) using robust full-information maximum likelihood estimation (Schafer and Graham, 2002; Yuan and Bentler, 2000). Across the entire dataset, 2.86% of response data was missing. Following the customary procedure, we analyzed model fit by means of the *χ*^2^-test and common descriptive fit measures: the comparative fit index (*CFI*), the Tucker–Lewis index (*TLI*), the root mean square error of approximation (*RMSEA*), and the standardized root mean square residual (*SRMR*). Based on the typical recommendations, *CFI* and *TLI* should be equal to or greater than 0.90, and even better if it is equal to or greater than 0.95, while *RMSEA* and *SRMR* should be smaller than 0.08, and even better if it is equal or greater than 0.05 (Hu and Bentler, 1999; Schermelleh-Engel et al., 2003). We considered any given model fit as satisfactory if at least three out of the four indices fulfilled the criteria. Moreover, we report omega as a measure of internal consistency. Since the multiple factors in our model are only moderately correlated and no second-order or general construct can be assumed, we utilized McDonald’s (1999) basic formula. Finally, we tested the model for measurement invariance across age groups (two groups, split on the median) and participant gender. To this end, we used the common procedure described by Meredith (1993) of successively constraining factor loadings, item intercepts, and item residual variances. We utilized the common between-model cutoffs of 0.010 for *CFI* and 0.015 for *RMSEA* (Cheung and Rensvold, 2002; Chen, 2007).

## Results

### Sample characteristics

We analyzed the responses of participants speaking various languages (Chinese simplified, Chinese traditional, English, Farsi, Finnish, German, Icelandic, Italian, Portuguese, Spanish, and Turkish) around the world. The total sample comprised of *N* = 15,693 individuals. In general, more women than men participated in the study, with Iceland having, descriptively, the highest ratio of female participation (78.7%). The age average for the various samples ranged between 25 and 42 years. Details of the sample are provided in Table 1.

**Table 1:** Sample characteristics.

Language	*n*	Margin of error, %	Female (*n*, %)	Age (*M* ± *SD*)
Chinese (simplified)	922	3	482, 53.6%	25.18 ± 9.04
Chinese (traditional)	1,172	3	621, 53.3%	36.17 ± 15.38
English	7,120	1	4,285, 6.5%	33.04 ± 13.14
Farsi	194	7	121, 63%	34.21 ± 9.85
Finnish	220	7	137, 62.3%	42.64 ± 11.31
German	2,599	2	1,629, 63.1%	37.29 ± 14.47
Icelandic	423	5	333, 78.7%	42.79 ± 12.76
Italian	1,324	3	668, 5.6%	39.56 ± 16.42
Portuguese	553	4	353, 64.3%	34.8 ± 11.58
Spanish	541	4	265, 49.3%	31.74 ± 12.43
Turkish	625	4	380, 61.5%	30.78 ± 21.23

### Factorial validity

We computed a CFA with the above-mentioned items in a correlated factors model with four latent constructs. The majority of the models exhibited a good fit, except for the Chinese (traditional) and Turkish (see Table 2). Similarly, the majority of different language versions showed good to very good reliability for the subscales—except for the subscale *Vigor* in English, Icelandic, Portuguese, and Spanish. All standardized factor loadings were equal to or greater than 0.744. At the behest of a reviewer, we added exploratory factor analyses to check the suitability of different numbers of factors in different language versions (see Supplementary material). While 1 out of the 11 language versions could potentially be reduced to 3 factors, all 10 other versions required at least 4 factors. Thus, in the interest of cross-cultural comparability and unity of the instrument, we decide in favor of a uniform four-factor solution.

**Table 2:** Model fit of each assessed language.

Language	χ^2^	df	*p*	CFI	TLI	RMSEA	Lower CI	Upper CI	SRMR	Ω			
										Fatigue	Vigor	Anger	Dejection
Chinese (simplified)	645.253	98	<0.001	0.918	0.900	0.091	0.084	0.098	0.088	0.867	0.744	0.834	0.854
Chinese (traditional)	948.78	98	<0.001	0.895	0.872	0.103	0.097	0.109	0.098	0.869	0.724	0.783	0.836
English	3197.312	98	<0.001	0.921	0.903	0.073	0.071	0.076	0.065	0.861	0.619	0.803	0.789
Farsi	212.764	98	<0.001	0.923	0.905	0.085	0.068	0.101	0.061	0.803	0.821	0.836	0.856
Finnish	177.126	98	<0.001	0.951	0.940	0.065	0.049	0.081	0.053	0.900	0.818	0.858	0.817
German	1088.092	98	<0.001	0.950	0.938	0.070	0.066	0.074	0.052	0.886	0.873	0.876	0.818
Icelandic	223.829	98	<0.001	0.961	0.952	0.059	0.049	0.070	0.044	0.915	0.695	0.843	0.833
Italian	801.733	98	<0.001	0.915	0.896	0.083	0.078	0.088	0.061	0.833	0.881	0.82	0.785
Portuguese	275.634	98	<0.001	0.952	0.941	0.064	0.055	0.073	0.063	0.906	0.664	0.87	0.808
Spanish	341.966	98	<0.001	0.921	0.904	0.075	0.067	0.084	0.088	0.859	0.579	0.872	0.755
Turkish	632.154	98	<0.001	0.901	0.879	0.105	0.097	0.113	0.077	0.899	0.764	0.814	0.763

We reported omega values for reliability.

### Measurement invariance

Finally, we tested each language version for measurement invariance across participant gender and age groups. For the age groups, we split each language sample at the median. We report the results of the step-wise test process in Tables 3, 4. The model in each different language is strictly invariant across gender and age groups, with some exceptions. Concerning gender, the language version of Farsi did not exhibit strict invariance—only strong invariance. The other reported languages showed evidence for strict gender invariance. Regarding age, the Chinese, Farsi, Finish, and Turkish versions provide evidence for strict invariance. The remaining language versions did not show strict invariance but strong invariance—with the exception of the Italian version.

**Table 3:** Measurement invariance with regard to sex.

Language	*Model*	*χ^2^*	*Δχ^2^*	df	Δdf	*p*	CFI	ΔCFI	RMSEA	ΔRMSEA
Chinese (simplified)	Configural	775.724		196			0.917		0.092	
Chinese (simplified)	Metric	792.918	17.194	208	12	0.142	0.915	0.002	0.090	0.002
Chinese (simplified)	Scalar	842.285	49.366	220	12	<0.001	0.911	0.004	0.090	0.000
Chinese (simplified)	Strict	881.231	38.947	236	16	0.001	0.906	0.005	0.089	0.001
Chinese (traditional)	Configural	1044.389		196			0.892		0.104	
Chinese (traditional)	Metric	1068.289	23.900	208	12	0.021	0.892	0.001	0.101	0.003
Chinese (traditional)	Scalar	1096.357	28.068	220	12	0.005	0.891	0.001	0.099	0.003
Chinese (traditional)	Strict	1091.611	4.747	236	16	0.997	0.892	0.001	0.095	0.004
English	Configural	3225.091		196			0.921		0.073	
English	Metric	3275.085	49.994	208	12	<0.001	0.920	0.001	0.071	0.002
English	Scalar	3422.025	146.939	220	12	<0.001	0.917	0.003	0.071	0.001
English	Strict	3508.287	86.262	236	16	<0.001	0.915	0.003	0.069	0.001
Farsi	Configural	32.397		196			0.924		0.084	
Farsi	Metric	333.141	12.744	208	12	0.388	0.922	0.002	0.082	0.001
Farsi	Scalar	34.362	7.221	220	12	0.843	0.925	0.004	0.078	0.004
Farsi	Strict	373.031	32.669	236	16	0.008	0.914	0.011	0.081	0.003
Finnish	Configural	307.207		196			0.934		0.074	
Finnish	Metric	319.437	12.230	208	12	0.427	0.933	0.001	0.072	0.002
Finnish	Scalar	332.579	13.142	220	12	0.359	0.933	0.000	0.070	0.002
Finnish	Strict	333.966	1.387	236	16	1.000	0.939	0.006	0.065	0.006
German	Configural	1238.140		196			0.947		0.072	
German	Metric	1237.566	0.574	208	12	1.000	0.947	0.000	0.069	0.002
German	Scalar	130.445	62.878	220	12	<0.001	0.945	0.002	0.069	0.001
German	Strict	1353.192	52.747	236	16	<0.001	0.941	0.004	0.069	0.000
Icelandic	Configural	397.080		196			0.949		0.068	
Icelandic	Metric	40.556	3.476	208	12	0.991	0.948	0.001	0.066	0.002
Icelandic	Scalar	412.861	12.305	220	12	0.422	0.949	0.001	0.064	0.002
Icelandic	Strict	413.867	1.006	236	16	1.000	0.948	0.001	0.062	0.002
Italian	Configural	867.084		196			0.919		0.081	
Italian	Metric	892.221	25.137	208	12	0.014	0.918	0.002	0.079	0.001
Italian	Scalar	948.548	56.327	220	12	<0.001	0.913	0.005	0.079	0.000
Italian	Strict	1011.600	63.052	236	16	<0.001	0.906	0.007	0.079	0.000
Portuguese	Configural	357.187		196			0.958		0.059	
Portuguese	Metric	378.371	21.184	208	12	0.048	0.956	0.002	0.059	0.000
Portuguese	Scalar	401.396	23.026	220	12	0.028	0.954	0.003	0.059	0.000
Portuguese	Strict	425.607	24.211	236	16	0.085	0.950	0.003	0.059	0.000
Spanish	Configural	467.069		196			0.916		0.077	
Spanish	Metric	479.648	12.580	208	12	0.400	0.913	0.004	0.077	0.001
Spanish	Scalar	498.411	18.763	220	12	0.094	0.911	0.002	0.075	0.001
Spanish	Strict	533.878	35.467	236	16	0.003	0.904	0.008	0.076	0.000
Turkish	Configural	765.972		196			0.895		0.108	
Turkish	Metric	796.985	31.013	208	12	0.002	0.892	0.003	0.107	0.002
Turkish	Scalar	834.690	37.705	220	12	<0.001	0.888	0.004	0.105	0.001
Turkish	Strict	856.856	22.166	236	16	0.138	0.886	0.002	0.103	0.003

**Table 4:** Measurement invariance with regard to age.

Language	Model	*χ*^2^	Δ*χ*^2^	df	Δdf	*p*	CFI	ΔCFI	RMSEA	ΔRMSEA
Chinese (simplified)	Configural	782.449		196			0.917		0.091	
Chinese (simplified)	Metric	795.391	12.942	208	12	0.373	0.917	0.000	0.089	0.003
Chinese (simplified)	Scalar	822.581	27.191	220	12	0.007	0.916	0.001	0.087	0.002
Chinese (simplified)	Strict	828.243	5.662	236	16	0.991	0.915	0.001	0.084	0.003
Chinese (traditional)	Configural	1064.389		196			0.894		0.103	
Chinese (traditional)	Metric	1106.218	41.829	208	12	<0.001	0.891	0.002	0.101	0.002
Chinese (traditional)	Scalar	1156.406	5.189	220	12	<0.001	0.888	0.003	0.100	0.001
Chinese (traditional)	Strict	1186.872	3.466	236	16	0.016	0.884	0.004	0.098	0.002
English	Configural	3474.126		196			0.916		0.076	
English	Metric	3531.847	57.721	208	12	<0.001	0.915	0.001	0.074	0.002
English	Scalar	3682.031	15.184	220	12	<0.001	0.912	0.003	0.073	0.001
English	Strict	4292.368	61.337	236	16	<0.001	0.895	0.017	0.077	0.004
Farsi	Configural	407.490		196			0.871		0.110	
Farsi	Metric	424.371	16.881	208	12	0.154	0.867	0.004	0.108	0.001
Farsi	Scalar	438.834	14.463	220	12	0.272	0.866	0.001	0.106	0.003
Farsi	Strict	448.674	9.840	236	16	0.875	0.868	0.002	0.101	0.004
Finnish	Configural	366.456		196			0.912		0.089	
Finnish	Metric	375.606	9.150	208	12	0.690	0.913	0.001	0.086	0.003
Finnish	Scalar	391.541	15.935	220	12	0.194	0.911	0.001	0.084	0.002
Finnish	Strict	405.706	14.165	236	16	0.586	0.911	0.001	0.081	0.003
German	Configural	1164.390		196			0.952		0.069	
German	Metric	1192.162	27.772	208	12	0.006	0.951	0.001	0.067	0.001
German	Scalar	1407.062	214.900	220	12	<0.001	0.941	0.010	0.071	0.004
German	Strict	1788.009	38.947	236	16	<0.001	0.921	0.020	0.080	0.009
Icelandic	Configural	361.055		196			0.954		0.064	
Icelandic	Metric	393.531	32.476	208	12	0.001	0.949	0.006	0.065	0.002
Icelandic	Scalar	422.732	29.201	220	12	0.004	0.944	0.005	0.066	0.001
Icelandic	Strict	476.488	53.756	236	16	<0.001	0.926	0.018	0.074	0.007
Italian	Configural	87.956		196			0.919		0.081	
Italian	Metric	928.266	57.310	208	12	<0.001	0.914	0.005	0.081	0.000
Italian	Scalar	1105.393	177.127	220	12	<0.001	0.895	0.019	0.087	0.006
Italian	Strict	1255.493	15.101	236	16	<0.001	0.878	0.017	0.090	0.004
Portuguese	Configural	416.917		196			0.943		0.069	
Portuguese	Metric	43.341	13.424	208	12	0.339	0.943	0.000	0.067	0.002
Portuguese	Scalar	452.105	21.764	220	12	0.040	0.940	0.002	0.066	0.001
Portuguese	Strict	51.810	58.705	236	16	<0.001	0.928	0.013	0.070	0.004
Spanish	Configural	504.322		196			0.905		0.083	
Spanish	Metric	524.940	2.618	208	12	0.056	0.903	0.002	0.081	0.002
Spanish	Scalar	563.295	38.355	220	12	<0.001	0.895	0.008	0.082	0.001
Spanish	Strict	689.575	126.280	236	16	<0.001	0.860	0.036	0.092	0.010
Turkish	Configural	733.018		196			0.904		0.103	
Turkish	Metric	745.663	12.645	208	12	0.395	0.904	0.000	0.100	0.003
Turkish	Scalar	788.416	42.753	220	12	<0.001	0.899	0.005	0.100	0.000
Turkish	Strict	844.341	55.925	236	16	<0.001	0.890	0.009	0.101	0.001

## Discussion

The Profile of Mood States questionnaire is a widely utilized instrument in psychological research. Its applications range from basic research on cognitive issues to applied fields such as work psychology, athletics, and clinical settings. The study at hand aimed to assess the psychometric properties of the newly developed POMS-16 across 10 different languages. Specifically, we aimed to investigate whether the established four-factor structure of the instrument could be replicated in the languages previously described, while also examining measurement invariance across age and gender. Overall, the results of the factor analysis indicated a satisfactory fit for the majority of languages, with the exception of Chinese (traditional) and Turkish versions. However, even these versions with slightly worse fit may prove useful depending on their intended use case. Additionally, the majority of language versions demonstrated strong evidence of reliability across all subscales, although exceptions were observed for the *Vigor* subscale in the English, Icelandic, Portuguese, and Spanish versions. Regarding measurement invariance, the model for each language displayed evidence of strict invariance across gender and age groups, although some exceptions were noted.

### Factorial structure

In detail, compared to the other language versions, the Chinese (traditional) and Turkish versions did not reveal a satisfactory fit. To date, the shortest Chinese version (Chen et al., 2002) comprises 30 items and was validated by showing excellent reliability and a one-factor structure, which makes it not comparable to our findings of a four-factor structure. In comparison to our scale, Chen et al. (2002) investigated a Taiwanese-speaking elderly community (i.e., aged 65 years and above). Similarly, there is no short version of the Turkish language version (Selvi et al., 2011). The latter replicated the original six-factor solution containing 58 items. Therefore, there is a need for further studies in order to verify our initial results of the short Turkish version. The data of the following language versions English, Farsi, Finnish, German, Icelandic, Italian, Portuguese, and Spanish provided evidence of a satisfactory fit model. It is worth noting that some studies have used an English version for Finish athletes (Heikura et al., 2023; Huttunen et al., 2004) or a modified version of the POMS, as applied by Azizi et al. (2021) in Iran during COVID-19. However, there are no official translations nor validity studies for the assessment of POMS in Farsi, Finish, or Icelandic. Consequently, the present instrument provides a valid short measure for the evaluation of mood states in these languages.

Furthermore, our revealed factor structure differs from the results provided by Cella et al. (1987), which represents the shortest English version available. In contrast to our studied population, it was validated in a cancer patient population, illustrating a one-factor model without the subscale *Vigor*. The German version in this study validates past results by Petrowski et al. (2021) showing a four-factorial structure and similar psychometric properties. The Italian short version of the POMS (Mannarini et al., 2012) shows a two-factor structure with 13 items, which was also evaluated in a patient sample with cancer. The authors renamed the scale to “Negative and Positive Mood State Short Form,” leaving out a differentiation of broader constructs, as depicted in the German version (Petrowski et al., 2021). Furthermore, our findings also differ from those provided in the Portuguese version of POMS (Pereira et al., 2023). Compared to our sample, the authors evaluated a short version based on student population, exhibiting a three-factor structure (i.e., depression, hostility, and vigor) encompassing 12 items. Finally, a short Spanish version (Andrade et al., 2010) demonstrated a five-factor structure with 30 items, validated in an athlete sample. Although shorter than the original, our newly validated Spanish version appears more suitable for purposes that require frequent and rapid self-monitoring.

### Measurement invariance

To the best of our knowledge, the analysis of the present study provides the first evidence of measurement invariance in the reported languages with regard to gender and age. However, measurement invariance of the short German version of the POMS-16 has been previously assessed (Petrowski et al., 2021). The current results replicate past findings revealing gender invariance and strong evidence for age invariance. With the exception of Farsi, all of the other reported languages exhibited evidence for strict gender invariance, although Farsi was still strongly invariant. The Chinese, Farsi, Finish, and Turkish versions were strictly invariant across ages. The remaining versions did not show strict invariance for age, except for the Italian version, which supported strong invariance for age. In practice, strong invariance is considered sufficient to allow for valid comparisons between groups (Gregorich, 2006; Schmalbach and Zenger, 2019). In sum, the findings of this study have implications for both research and clinical practice. Overall, the POMS-16 questionnaire demonstrates satisfactory fit and strong reliability across most languages, indicating its validity for assessing mood states in diverse cultural contexts. However, exceptions in the *Vigor* subscale for certain language versions suggest the need for cautious interpretation and potential adaptation of the instrument. Despite this, measurement invariance across gender and age groups was generally supported, enhancing the utility of the POMS-16 for comparative research and clinical assessments. Additionally, the POMS in diverse language versions expands its accessibility and applicability, facilitating cross-cultural research and improving assessment accuracy in clinical settings. Future research should consider using the POMS for comparisons between the different cultures and countries examined in the present research.

### Limitations

Data collection was carried out during the initial COVID lockdowns in the spring of 2020. Accordingly, it should be noted that this was quite a unique time, which was likely to have an influence on respondents’ moods. However, it remains unclear whether the evaluation process itself, via the questionnaire, was affected by this. Despite this, the overall psychometric results are comparable to those of previous research, without considering this special circumstance.

## Conclusion

The present study aimed to assess the psychometric properties of the POMS-16 questionnaire across 10 languages, examining its four-factor structure and measurement invariance across age and gender. The results indicate a satisfactory fit for the majority of languages, except the Chinese (traditional) and Turkish versions. The majority of language versions showed strong reliability across subscales, with exceptions in the *Vigor* subscale for English, Icelandic, Portuguese, and Spanish versions. Measurement invariance was generally supported across gender and age groups, although some exceptions were noted. Notably, we provided the first version of the POMS in Farsi, Finnish, and Icelandic. These findings enhance the cross-cultural applicability of the POMS-16, contributing to its utility in diverse populations and thus enhancing the comparability of the results.

## Data Availability

The raw data supporting the conclusions of this article will be made available by the authors, without undue reservation.
