# Supplementary material for: Cross-cultural validation of the profile of mood scale: evaluation of the psychometric properties of short screening versions
Source: Front Psychol. 2025 Mar 17;16:1498717. doi: 10.3389/fpsyg.2025.1498717 (PMC11955696; doi:10.3389/fpsyg.2025.1498717)
Supplement: Supplementary file 1 [file Table_1.docx]

Supplementary Material

# Exploratory factor analyses

In order to explore different potential configurations and factor numbers, we conducted parallel analysis on the different language versions of the POMS. To this end, we utilized the *fa.parallel* function of the *psych* package using OLS exploratory factor analysis and comparing the extracted empirical eigenvalues to the 95^th^ percentile of the simulated eigenvalue distribution. As per Table S1, 10 out of the 11 tested language versions of the POMS are ideally represented by four or more factors. Only the Farsi version could potentially be reduced to just three factors. Tables S2.1 to S2.11 contain the results of the individual analyses.

We then tested the model fit for the Farsi version of the POMS, once using the four-factorial configuration that was used for all other languages as well, and once using a reconfigured three-factorial model as displayed in Table S3 combining the Fatigue and Dejection factors. The former had barely acceptable fit as reported in the main manuscript, χ²(98) = 212.76, *p* < .001, *CFI* = .923, *TLI* = .905, *RMSEA* = .085, *SRMR* = .061. By comparison, the three-factorial model had slightly improved fit, χ²(101) = 183.35, *p* < .001, *CFI* = .941, *TLI* = .930, *RMSEA* = .073, *SRMR* = .060. In our view, this slight improvement in model fit does not warrant a departure from the unified POMS-16 questionnaire. Cross-country and -culture comparability should be valued highly and the aim of the present study is to check whether the POMS-16 as originally developed can be considered acceptable in these various languages.

Table S1

*Parallel analysis results*

| Language | Number of factors |
| --- | --- |
| Chinese (simplified) | 5 |
| Chinese (traditional) | 6 |
| English | 7 |
| German | 4 |
| Farsi | 3 |
| Finnish | 4 |
| Icelandic | 4 |
| Italian | 4 |
| Portuguese | 4 |
| Spanish | 4 |
| Turkish | 4 |

Table S2.1

Parallel analysis of the Chinese (simplified) POMS

| Number of Factors | Empirical eigenvalues | 95th percentile of simulated eigenvalues |
| --- | --- | --- |
| 1 | 7.13950720 | 0.37439909 |
| 2 | 1.18260909 | 0.20195296 |
| 3 | 0.44536118 | 0.16662086 |
| 4 | 0.19780237 | 0.13247560 |
| 5 | 0.13486436 | 0.10425230 |
| 6 | 0.03712778 | 0.07526674 |
| 7 | -0.01165965 | 0.04797572 |
| 8 | -0.05539206 | 0.02404349 |

Table S2.2

Parallel analysis of the Chinese (traditional) POMS

|  |
| --- |

| Number of Factors | Empirical eigenvalues | 95th percentile of simulated eigenvalues |
| --- | --- | --- |
| 1 | 6.880398845 | 0.29854864 |
| 2 | 1.307555407 | 0.17624049 |
| 3 | 0.422740565 | 0.14438016 |
| 4 | 0.282882023 | 0.11478498 |
| 5 | 0.170557998 | 0.09046045 |
| 6 | 0.099013853 | 0.06749594 |
| 7 | 0.004650915 | 0.04354066 |
| 8 | -0.052245879 | 0.02210778 |

Table S2.3

Parallel analysis of the English POMS

|  |
| --- |

| Number of Factors | Empirical eigenvalues | 95th percentile of simulated eigenvalues |
| --- | --- | --- |
| 1 | 5.71033234 | 0.203103760 |
| 2 | 1.01204850 | 0.068775230 |
| 3 | 0.44604836 | 0.057791183 |
| 4 | 0.24419332 | 0.046622447 |
| 5 | 0.11369596 | 0.036107024 |
| 6 | 0.06335615 | 0.027171972 |
| 7 | 0.03174998 | 0.018237368 |
| 8 | -0.03602006 | 0.009551578 |

Table S2.4

Parallel analysis of the German POMS

|  |
| --- |

| Number of Factors | Empirical eigenvalues | 95th percentile of simulated eigenvalues |
| --- | --- | --- |
| 1 | 6.584668146 | 0.26604800 |
| 2 | 1.482212337 | 0.11480872 |
| 3 | 0.749427084 | 0.09489637 |
| 4 | 0.387102952 | 0.07691752 |
| 5 | 0.006593373 | 0.06034048 |
| 6 | -0.052258716 | 0.04369435 |
| 7 | -0.123250206 | 0.02959994 |
| 8 | -0.161047286 | 0.01541924 |

Table S2.5

Parallel analysis of the Farsi POMS

|  |
| --- |

| Number of Factors | Empirical eigenvalues | 95th percentile of simulated eigenvalues |
| --- | --- | --- |
| 1 | 7.28974760 | 0.66849468 |
| 2 | 1.01290959 | 0.44141581 |
| 3 | 0.48284131 | 0.35441332 |
| 4 | 0.22381664 | 0.28344577 |
| 5 | 0.19087007 | 0.21556221 |
| 6 | 0.03607289 | 0.15679860 |
| 7 | 0.01620009 | 0.09606791 |
| 8 | -0.06717299 | 0.03605554 |

Table S2.6

Parallel analysis of the Finnish POMS

|  |
| --- |

| Number of Factors | Empirical eigenvalues | 95th percentile of simulated eigenvalues |
| --- | --- | --- |
| 1 | 6.199724158 | 0.66296208 |
| 2 | 1.145453856 | 0.40768875 |
| 3 | 0.943324281 | 0.33049155 |
| 4 | 0.427403602 | 0.25940335 |
| 5 | 0.136635346 | 0.19927518 |
| 6 | -0.009907143 | 0.14335970 |
| 7 | -0.058423611 | 0.08670709 |
| 8 | -0.132730210 | 0.03996053 |

Table S2.7

Parallel analysis of the Icelandic POMS

|  |
| --- |

| Number of Factors | Empirical eigenvalues | 95th percentile of simulated eigenvalues |
| --- | --- | --- |
| 1 | 6.678703206 | 0.48948642 |
| 2 | 1.055675774 | 0.29644455 |
| 3 | 0.619918185 | 0.23871254 |
| 4 | 0.251108483 | 0.18932638 |
| 5 | 0.055939242 | 0.14313773 |
| 6 | -0.004095108 | 0.10742490 |
| 7 | -0.030684507 | 0.06949846 |
| 8 | -0.063289093 | 0.03012563 |

Table S2.8

Parallel analysis of the Italian POMS

|  |
| --- |

| Number of Factors | Empirical eigenvalues | 95th percentile of simulated eigenvalues |
| --- | --- | --- |
| 1 | 5.969000353 | 0.31563377 |
| 2 | 1.497399563 | 0.16592735 |
| 3 | 0.617690841 | 0.13469952 |
| 4 | 0.239172240 | 0.10975411 |
| 5 | 0.084820331 | 0.08585233 |
| 6 | 0.009886328 | 0.06243412 |
| 7 | -0.058790630 | 0.03947107 |
| 8 | -0.116959551 | 0.01973059 |

Table S2.9

Parallel analysis of the Portuguese POMS

|  |
| --- |

| Number of Factors | Empirical eigenvalues | 95th percentile of simulated eigenvalues |
| --- | --- | --- |
| 1 | 6.13752663 | 0.43629080 |
| 2 | 0.99270526 | 0.25948582 |
| 3 | 0.89669571 | 0.21627923 |
| 4 | 0.44922214 | 0.17175475 |
| 5 | 0.02271799 | 0.13117885 |
| 6 | -0.02854845 | 0.09551156 |
| 7 | -0.06348286 | 0.05948667 |
| 8 | -0.11345686 | 0.02656718 |

Table S2.10

Parallel analysis of the Spanish POMS

|  |
| --- |

| Number of Factors | Empirical eigenvalues | 95th percentile of simulated eigenvalues |
| --- | --- | --- |
| 1 | 5.927655730 | 0.43511396 |
| 2 | 0.852324773 | 0.26341356 |
| 3 | 0.661053974 | 0.21549648 |
| 4 | 0.218627311 | 0.17003756 |
| 5 | 0.108439598 | 0.13227488 |
| 6 | 0.026464556 | 0.09443500 |
| 7 | 0.003193857 | 0.06564794 |
| 8 | -0.046056882 | 0.02949078 |

Table S2.11

Parallel analysis of the Turkish POMS

|  |
| --- |

| Number of Factors | Empirical eigenvalues | 95th percentile of simulated eigenvalues |
| --- | --- | --- |
| 1 | 7.87218616 | 0.43191168 |
| 2 | 0.85789677 | 0.24455081 |
| 3 | 0.44781847 | 0.19875832 |
| 4 | 0.19501666 | 0.15807979 |
| 5 | 0.13046328 | 0.12321101 |
| 6 | 0.07747252 | 0.09014795 |
| 7 | -0.01435482 | 0.05786209 |
| 8 | -0.02924410 | 0.02830156 |

Table S3

Normal and alternate factor structure for the Farsi POMS-16

|  |
| --- |

|  | Four-factor model | | | | | Three-factor model (Farsi) | | |
| --- | --- | --- | --- | --- | --- | --- | --- | --- |
| Item | Fatigue | Vigor | Anger | Dejection |  | Fatigue/Dejection | Vigor | Anger |
| 1 |  |  | X |  |  |  |  | X |
| 2 | X |  |  |  |  | X |  |  |
| 3 |  | X |  |  |  |  | X |  |
| 4 |  |  |  | X |  | X |  |  |
| 5 |  |  | X |  |  |  |  | X |
| 6 |  |  |  | X |  | X |  |  |
| 7 | X |  |  |  |  | X |  |  |
| 8 |  |  | X |  |  | X |  |  |
| 9 | X |  |  |  |  | X |  |  |
| 10 |  |  |  | X |  | X |  |  |
| 11 | X |  |  |  |  | X |  |  |
| 12 |  |  |  | X |  | X |  |  |
| 13 |  | X |  |  |  |  | X |  |
| 14 |  |  | X |  |  |  |  | X |
| 15 |  | X |  |  |  |  | X |  |
| 16 |  | X |  |  |  |  | X |  |
